# Comprehensive Pan-Cancer Analyses of Pyroptosis-Related Genes to Predict Survival and Immunotherapeutic Outcome

**DOI:** 10.3390/cancers14010237

**Published:** 2022-01-04

**Authors:** Qilin Wang, Qian Liu, Sihan Qi, Junyou Zhang, Xian Liu, Xin Li, Chunyan Li

**Affiliations:** 1 School of Engineering Medicine, Beihang University, Beijing 100191, China; wangqilin@buaa.edu.cn (Q.W.); ZY1910219@buaa.edu.cn (Q.L.); zy2010120@buaa.edu.cn (S.Q.); Zhangjunyou@buaa.edu.cn (J.Z.); 2 School of Biological Science and Medical Engineering, Beihang University, Beijing 100191, China; 3 Beijing Advanced Innovation Center for Big Data-Based Precision Medicine, Beihang University, Beijing 100191, China; 4 State Key Laboratory of Molecular Developmental Biology, Institute of Genetics and Developmental Biology, Chinese Academy of Sciences, Beijing 100101, China; xliu15@genetics.ac.cn (X.L.); xinli@genetics.ac.cn (X.L.); 5 Key Laboratory of Big Data-Based Precision Medicine (Ministry of Industry and Information Technology), Beihang University, Beijing 100191, China

**Keywords:** pyroptosis, pan-cancer, tumor microenvironment, immune therapy

## Abstract

**Simple Summary:**

Pyroptosis is a type of programmed cell death accompanied by inflammation. Although the dysregulation of pyroptosis has been reported to be involved in carcinogenesis, its function in cancer progression and therapy remains largely unknown and controversial because of the inconsistency across different cancer types. This study provides the most complete gene set of pyroptosis-related genes (PRGs), depicts their expression changes across 31 cancer types for the first time, and constructs a novel prognostic risk model to predict cancer patient survival. In addition, the effects of pyroptosis on immune cell infiltration and immunotherapy were dissected at the pan-cancer level. Small-molecule compounds, which may be beneficial to immunotherapy, were screened on the basis of differentially expressed PRGs. These results lay the foundation for the study of pyroptosis in cancer.

**Abstract:**

Pyroptosis is a newly characterized type of programmed cell death. However, its function in cancer progression and its response to treatments remain controversial. Here, we extensively and systematically compiled genes associated with pyroptosis, integrated multiomics data and clinical data across 31 cancer types from The Cancer Genome Atlas, and delineated the global alterations in PRGs at the transcriptional level. The underlying transcriptional regulations by copy number variation, miRNAs, and enhancers were elucidated by integrating data from the Genotype-Tissue Expression and International Cancer Genome Consortium. A prognostic risk model, based on the expression of PRGs across 31 cancer types, was constructed. To investigate the role of pyroptosis in immunotherapy, we found five PRGs associated with effectiveness by exploring the RNA-Seq data of patients with immunotherapy, and further identified two small-molecule compounds that are potentially beneficial for immunotherapy. For the first time, from a pyroptosis standpoint, this study establishes a novel strategy to predict cancer patient survival and immunotherapeutic outcomes.

## 1. Introduction

**Figure 1:**
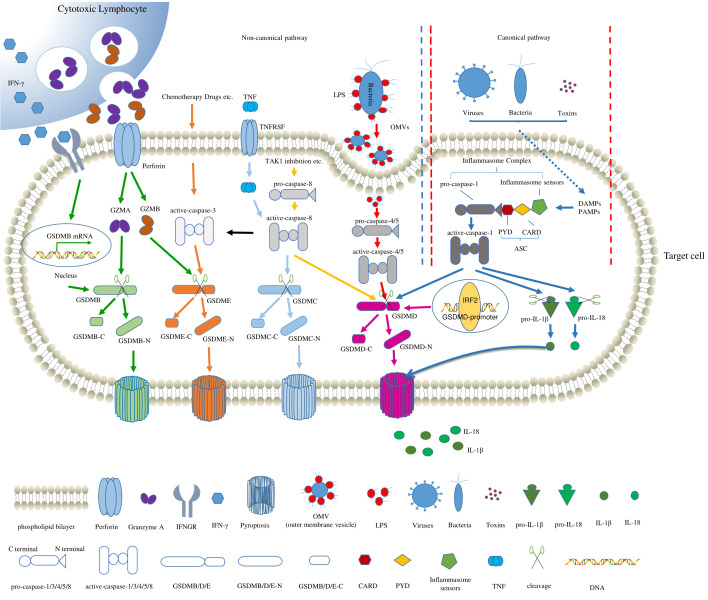
Schematic diagram of pyroptosis. The representative PRGs are depicted in the canonical and noncanonical pathways. For the canonical pathway (within the red dotted lines), caspase-1, activated by inflammasomes, cleaves GSDMD to release GSDMD-N, which accumulates on the cell membrane to perforate and cause pyroptosis. The inflammasome consists of an inflammasome sensor, ASC, and an effector (pro-caspase-1). Moreover, caspase-1 promotes the maturation and secretion of proinflammatory factors (IL-1β and IL-18), which leads to the expansion of the inflammatory response. For the noncanonical pathway (left to the blue dotted lines), pyroptosis is mainly mediated by caspase-4/5, which cleaves GSDMD to induce pyroptosis. In addition, caspase-3/8 cleaves GSDME, and lymphocyte-derived granzyme A (GZMA) cleaves GSDMB to induce pyroptosis. The α-ketoglutarate increases the ROS levels to induce the oxidation and internalization of the plasma-membrane-localized death receptor, DR6, which recruits and activates both pro-caspase-8 and GSDMC to induce pyroptosis.

Pyroptosis is a programmed cell death process that was first described when primary mouse macrophages were treated with an anthrax lethal toxin [1,2,3,4,5,6]. It was mistakenly reported as apoptosis when the macrophages were infected with Shigella flexneri in 1992 [7]. In 2001, Brennan and Cookson first proposed “pyroptosis” as a mode of cell death induced by caspase-1 that could rapidly destroy the integrity of the cell membrane. Pyroptosis is accompanied by an inflammatory process [8]. In 2015, gasdermin D (GSDMD) was reported as the executor of pyroptosis for the first time [9]. Pyroptosis is characterized by a continuous expansion until the cell membrane ruptures, which leads to the release of the cell contents and activates strong inflammatory responses [10]. In general, pyroptosis is divided into the canonical pathway and the noncanonical pathway (Figure 1). An increasing number of genes have been reported to be involved in pyroptosis [9,11,12,13,14,15,16,17,18,19,20,21,22,23,24]. However, to date, pyroptosis-related genes (PRGs) are rarely collected in databases; for example, pyroptosis is not included in the Kyoto Encyclopedia of Genes and Genomes (KEGG) database, and only four genes are included in the REACTOME and Gene Ontology databases. The lack of systematically summarized PRGs hinders the research on pyroptosis that is based on bioinformatics.

For mammals, pyroptosis is an important immune response of the body [25], and it plays an important role in the fight against infection [26]. Pyroptosis promotes the death of injured cells during the infection process [27]. In addition, it acts as an alarm for immune cells recruited to the infected site to promote the clearance of pathogens, thereby effectively protecting the body [8]. Because chronic inflammation is a critical factor during cancer progression, pyroptosis has been found to be associated with cancer [28]. The occurrence and metastasis of many cancer cells are accompanied by changes in pyroptosis, which may represent potential cancer therapeutic targets [29]. On the other hand, pyroptosis in cancer cells can inhibit cancer growth. Studies have shown that pyroptosis induction is effective in curing hematological cancer [30]. The NLRP1 inflammasome can activate the caspase-1-dependent pyroptosis pathway and induce glioma cell death [31]. In 2013, Zhang et al. found that the expression of *GSDME* in hepatocellular carcinoma (HCC) cells was lower than that in noncancerous cells. When the levels of the GSDME protein increased, the proliferation of HCC cells was suppressed [32]. Furthermore, the cumulative effect of the inflammasomes can form a microenvironment suitable for tumor cell growth and can play a role in promoting tumor growth [33]. In 2016, one study demonstrated that tumor growth can be promoted by the inflammasome and IL-1β pathways [34]. Therefore, pyroptosis is a double-edged sword when it comes to cancer, and it is dependent on the cancer type.

The induction of apoptosis has been leveraged and applied to eliminate cancer cells, but because of the acquisition of antiapoptotic properties, cancer cases may develop drug resistance after a period of apoptosis induction [35]. Pyroptosis induction may be a good strategy for cancer therapy because it not only overcomes the apoptosis resistance of cancer cells, but it also triggers antitumor immunity [36,37,38]. If certain PRGs are similarly expressed in multiple cancers, a similar strategy may be applied to detect and treat different cancer types. Therefore, it is urgent to systematically analyze the roles of pyroptosis in cancer progression and the therapeutic response at the pan-cancer level.

In this study, we integrated genes related to pyroptosis in recently published articles to provide the most comprehensive list of PRGs. We established a risk prediction model based on the relationship between the pyroptotic gene expression level and the survival time across 31 cancer types, which was downloaded from TCGA data (https://www.cancer.gov/about-nci/organization/ccg/research/structural-genomics/tcga, accessed on 15 July 2021). On the basis of the risk model, functional enrichment analyses were performed to explore the underlying mechanisms between pyroptosis and cancer progression. To further explore the role of pyroptosis in cancer treatment, integrative analyses were performed that were based on RNA-seq data prior to immunotherapy, and on the effectiveness in non-small cell lung cancer (NSCLC) and melanoma. A total of five PRGs were characterized to be highly associated with an immunotherapeutic response. By analyzing the effect of 93 different small-molecule compounds on gene expression, Gemcitabine and entinostat are characterized as effective promoters of the expression of the five PRGs above. These findings provide a new treatment strategy to improve immunotherapeutic outcomes. To the best of our knowledge, this is the first study to underscore the importance of pyroptosis to the prediction of cancer patient survival and the immune therapeutic response at the pan-cancer level.

## 2. Materials and Methods

### 2.1. Deep Exploration of PRGs Using GeneMANIA

The exploration of the pyroptosis gene set was performed primarily using the GeneMANIA database. GeneMANIA searches publicly available biological datasets and finds genes sharing functions on the basis of gene interactions, including protein and genetic interactions, pathways, coexpressions, colocalizations, and protein domain similarities. In the default mode, GeneMANIA weighs the predicted genes according to the results of the GO database: the higher the weight of the gene, the stronger the correlation.

The steps for using GeneMANIA to collect PRGs are as follows. First, one of the 24 genes obtained in the previous analysis, such as *CASP1* or *GSDMD*, was entered in the white dialog box in the upper left corner. The website automatically generates a gene map that is functionally related to the input gene according to the built-in algorithm. The genes were recorded on the map, and the remaining 23 genes were repeated. All the genes functionally related to the previous 24 PRGs were merged and were used as the original gene list for subsequent analysis.

### 2.2. Data Collection and Preprocessing

We systematically searched for publicly available gene expression datasets, together with clinical annotations, for different cancer types. The data were primarily downloaded from TCGA, ICGC, and Genotype-Tissue Expression (GTEx). The Level 3 RNA sequencing (RNA-seq) data and the corresponding clinical information for 31 cancers from TCGA were downloaded from the UCSC Xena website (https://xenabrowser.net/datapages/, accessed on 15 July 2021). For the TCGA dataset, the transcripts-per-kilobase-million (TPM) values were used to measure the gene expression, and the data were analyzed using the “limma” R package. The RNA-seq data and the clinical information on the BRCA cancer samples were obtained from the ICGC portal (https://dcc.icgc.org/projects/LIRI-JP, accessed on 20 July 2021). PAAD cancer data were obtained from GTEx (https://gtexportal.org/, accessed on 20 July 2021). The data from TCGA, GTEx and ICGC are publicly available. The current research follows TCGA, GTEx, and ICGC data access policies and publication guidelines. In addition to the above three datasets, we also included four sample cohorts from patients with immunotherapy for this study: GSE78220, GSE115821, GSE135222, and GSE126044. The RNA-seq data for drug treatments come from the GSE96760, GSE165548, and GSE131085 cohorts. The raw data from the count datasets generated by Illumina were downloaded from the Gene-Expression Omnibus (GEO; https://www.ncbi.nlm.nih.gov/geo/, accessed on 8 November 2021). All analyses were performed on the basis of the pertinent guidelines and regulations.

### 2.3. Pyroptosis-Related Gene Expression Analysis

First, for the original biological count data, preprocessing and normalization were the first steps towards addressing the gene expression. This process removes bias from the count data to ensure its uniformity and integrity. Next, when calculating expression changes, the gene expression uses the median gene expression of different tumor types or normal tissues. The calculation result is shown as log_2_(Foldchange). To further characterize the overall expression of different cancers and genes, we used a weighted expression method to calculate the overall expression score of genes. First, the expression of the same gene in different cancer types was normalized, and the proportion in different cancer types was calculated on the basis of the sum of the gene expression. The product of the two is the weight of the gene in this cancer. Then, for the same cancer, score = Σ (gene expression) × (weight coefficient).

### 2.4. Clinical Features Analysis of TCGA Data

The R package, “survival”, was used to assess the prognostic potential of pyroptosis among cancers. According to the disease-free survival analysis of the gene expression, a Kaplan-Meier analysis was performed.

### 2.5. Copy Number Variation (CNV) Analysis

The masked copy number segment file is a table that associates continuous chromosome fragments with the genome coordinates, the mean array intensity, and the number of probes bound to each fragment after removing the Y chromosome and the probes containing germline mutations (42). The heterozygosity and homozygosity of the amplification and deletion were included to evaluate the copy number alteration of each gene, of which over five percent were regarded as “high-frequency” CNVs. GISTIC2 and the R package, “maftools”, were used to identify the CNV regions and annotate the genes.

### 2.6. Quantification of eRNA Expression

Annotations of the enhancers were obtained from EnhancerDB (http://lcbb.swjtu.edu.cn/EnhancerDB/, accessed on 9 October 2021) and eRic (https://hanlab.uth.edu/eRic/m1, accessed on 9 October 2021). EnhancerDB is an online database that is used to examine regulatory relationships in the context of enhancers. The eRic database collects the whole eRNA expression profile across TCGA samples, as well as the eRNA clinical features, eRNA target genes, and eRNA drug responses [39]. Both EnhancerDB and eRic use H3K4me1 and H3K27ac marks for enhancer annotations, and we used coannotated enhancers in both datasets.

### 2.7. Prediction of Transcription Factors Binding to Enhancers

HACER (http://bioinfo.vanderbilt.edu/AE/HACER/, accessed on 9 October 2021) is a human activity enhancer atlas. A total of 1,676,284 enhancers in human cell lines were cataloged and annotated. In addition, HACER determined 772,902 combinations of transcription factors and enhancers on the basis of a reanalysis of the ChIP-Seq data in ENCODE and the integration of a large amount of data predicting chromatin interactions [40].

### 2.8. Hi-C and ChIP-Seq (Chromatin Immunoprecipitation Sequencing) Data Analysis

The Hi-C and ChIP-Seq data for different cell lines were collected from the Encyclopedia of DNA Elements (ENCODE). We used the MACS2 (v.2.2.6) peak finding algorithm to identify regions of ChIP-Seq enrichment. Default parameters were used throughout the process. Bam files of the ChIP-Seq experiments were used for visualization in IGV. We used Juicer to process the Hi-C data. Juicer is an open-source tool for analyzing Hi-C datasets [41].

### 2.9. Construction and Validation of a Prognostic Signature of PRGs

A univariate Cox analysis, based on mRNA expression and disease-free survival (DFS), was performed to screen PRGs with prognostic value. To minimize the risk of overfitting, a LASSO-penalized Cox regression analysis was applied to construct a prognostic model. The LASSO algorithm was used for variable selection and shrinkage with the “glmnet” R package. The independent variable in the regression was used for the standardized expression matrix of the PRGs and the demographic information (including age, gender, race, and cancer stage). The age variable is the true age of the patient. The gender, race, and cancer stages form corresponding classification matrices, according to the number of categories from the TCGA clinical data. The response variable is the DFS period and the status of the patients in the TCGA cohort. The patient’s risk score was calculated on the basis of the PRG expression level and its corresponding regression coefficient. The established formula is: score = esum (expression of each gene × corresponding coefficient). According to the median value of the risk score, patients are divided into a high-risk group and a low-risk group.

The Schoenfeld residual method was used to judge the proportional hazards assumption of the Cox proportional hazard regression analysis. The Cox regression can be performed only if the impact of the risk factors on survival does not change with time. We used the “cox.zph()” function in the R “survminer” package to calculate the Schoenfeld residual plot of each covariate over time. *p* > 0.05 is a variable that meets the proportional hazards assumption.

### 2.10. Functional Enrichment Analysis

We used the DAVID website to perform GO and KEGG analyses on genes related to the risk model. The analysis tool was the DAVID Functional Annotation Bioinformatics Microarray Analysis (https://david.ncifcrf.gov/, accessed on 20 December 2021). The R package, “GOplot”, was used to draw Figure 5A, and the Z-score was used to measure the risk score increase or decrease in the GO terms. The calculation method is Z-score = (positive gene number-negative gene number)/sqrt(gene number).

### 2.11. Immune Features Analysis

To examine the relationship between pyroptosis and the immune microenvironment, we computed the Pearson correlation between the gene expression and the immune parameters, including the immune cell fractions. A partial Spearman correlation was used to perform this association analysis, and the abundance of immune cells was calculated using CIBERSORT.

### 2.12. Differential Expression Analysis

For the RNA-seq data that were preprocessed and standardized, we used DESeq2 for the differential expression analysis. DESeq2 models the expression value and uses a scale factor to explain the difference in the library depth. Then, DESeq2 estimates the dispersion of the genes and reduces these estimates to generate a more accurate dispersion estimate, thereby modeling the read count. Finally, DESeq2 fits a model of negative binomial distribution and uses the Wald test or a likelihood ratio test for the hypothesis testing. Finally, we used the criteria of fold change > 1.5 and *P_adj_* < 0.05 as the critical values for screening.

### 2.13. Analysis of the Impact of Small-Molecule Compounds on Gene Expression

Transcriptome data under the effects of 93 small-molecule compounds were provided in GSE96760, and the cells processed using DMSO were regarded as the control group. Each file has a “CR” number, which represents a different batch. By collecting drug data after 24 h, the RNA-seq data of the cells under drug influence, and those processed using DMSO with the same “CR” numbers, were analyzed using DESeq2 to characterize differentially expressed genes in order to identify more drugs that may effectively promote gene expression and to ensure the credibility of the analytic results. We screened out five genes, according to their differential expression fold changes from large to small: *CASP1*, *NLRP1*, *NLRP3*, *IRF2*, and *PYCARD*. The differential expression fold change and the *p* value of the fourth-ranked gene were used to record the differential expression fold change and the *p* value of this drug. Hence, volcano plots of the 93 drugs were created using the x-coordinate representing the differential fold change of the drug, and the y-coordinate indicating the −log10 (*p* value) of the drug.

### 2.14. ROC Curve Calculation

The principle of ROC curve production is to set a number of different critical values for continuous variables, calculate the corresponding sensitivity and specificity at each critical value, and then use the sensitivity as the ordinate, with 1-specificity drawn as a curve on the abscissa. According to the relationship between gene expression and survival time, use the “surv_cutpoint” function of the “survminer” R package to determine the optimal cut-off expression value.

### 2.15. Statistical Analysis

The Student’s *t*-test was utilized to compare the gene expression between the tumor and normal tissues. The DFS between the different groups was compared by Kaplan-Meier analysis and a logrank test. Univariate and multivariate Cox regression analyses were applied to identify the independent predictors of DFS. All statistical analyses were performed using R software (Version 3.5.3) or SPSS (Version 23.0). If not specified above, a *p* value less than 0.05 was considered as significant, and all *p* values were two-tailed.

## 3. Results

### 3.1. Compilation of Genes Involved in Pyroptosis

To better characterize genes involved in the process of pyroptosis, we first identified the core components of pyroptosis: the caspase family and the GSDM family. All genes in these two families were searched in GeneCards (https://www.genecards.org/, accessed on 20 December 2021). Only genes with descriptions of the function of pyroptosis in the “Summaries for Gene” section were recruited as core factors of pyroptosis. Although *CASP3* was not explored in GeneCards using the method above, CASP3 cleaves GSDME to induce pyroptosis [42]. Therefore, *CASP3* was also considered a core factor on the gene list. On the other hand, although the functional description of *GSDMA* on GeneCards mentioned that it mediates the process of pyroptosis, this process is only present in mice, and not in humans [43]. Therefore, we did not select *GSDMA* for the gene list. On the basis of the standards above, nine genes (*CASP1*, *CASP3*, *CASP4*, *CASP5*, *CASP8*, *GSDMD*, *GSDME* (*DFNA5*), *GSDMC*, and *GSDMB*) were screened out as core factors in pyroptosis. In addition, one of the characteristics of pyroptosis is the release of IL-1β and IL-18 [9]; hence, these two genes (*IL-1β* and *IL-18*) were also included in the gene set.

Inflammasome formation is crucial to the canonical pathway of pyroptosis, which is composed of inflammasome sensors, apoptosis-associated speck-like protein-containing CARD (ASC), and effectors (pro-caspase-1) [11]. We searched using the keyword, “inflammasome”, on Reactome (https://reactome.org/, accessed on 21 December 2021), a widely used pathway database used to screen related genes on the basis of keywords. We obtained a list of genes involved in the formation of inflammasomes. To further enhance the relativity, the same pipeline was executed as was for the screening of the core factors. Therefore, another thirteen genes (*AIM2*, *NLRP1*, *NLRP2*, *NLRP3*, *NLRP6*, *NLRP7*, *NLRP9*, *NLRP12*, *NLRX1*, *NLRC4*, *NOD2*, *TLR4*, and *MEFV* (*Pyrin*)) were attached to the gene list of pyroptosis.

To further expand the gene set, genes that were reported to perform pyroptosis functions or regulate the expression/function of the above genes were also added to the gene list. First, we used the website, GeneMANIA (http://genemania.org/search/homo-sapiens/, accessed on 21 December 2021), to search for genes that may be involved in the pyroptosis process, and we used them as the raw gene list for the next analysis. Second, we searched for, “Gene Name + pyroptosis”, in the PubMed database of NCBI, and only selected genes directly involved in pyroptosis or that regulate the expression or function of pyroptotic genes. After collation of the above factors, we obtained a list of 43 PRGs (Table S1). The representatives of these 43 genes are illustrated in Figure 1 in order to show their functions and crosstalk during pyroptosis.

### 3.2. Aberrant Expression of PRGs in Cancer

**Figure 2:**
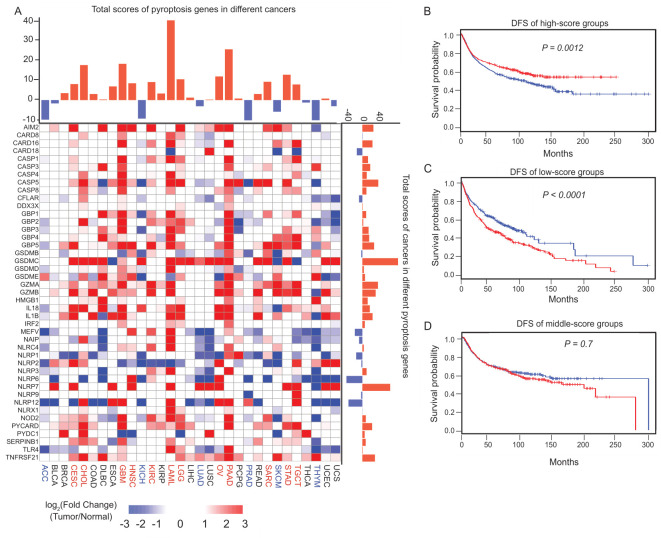
Pan-cancer analysis of PRGs. (**A**) The bars of the upper panel show the sums of different multiples of differentially expressed genes, and the heatmap shows the fold changes of PRGs (Tumor/Normal) in each cancer type (lower panel). The bars on the right of the lower panel show the sums of different multiples of genes across different cancer types. The names of the cancer types are in red for the high-score group, in blue for the low-score group, and in black for the middle-score group. Significantly upregulated and downregulated genes are marked in red and blue, respectively. (**B**–**D**) Kaplan-Meier curves for the disease-free survival (DFS) of samples in the high-score (**B**), low-score (**C**), and the middle-score (**D**) groups.

To determine the alterations of PRGs in cancer, we used The Cancer Genome Atlas (TCGA) data to determine the expression changes of PRGs in more than 10,000 cancer cases. Since there were no noncancerous data for MESO (mesothelioma) or UVM (uveal melanoma), the remaining 31 cancer types were further analyzed in this study. The abbreviations for the cancer types and the numbers of cancers and normal samples are shown in Table S2. To investigate the alterations in the gene expression patterns, a differential expression analysis was performed between cancer and normal tissues by cancer type, which demonstrated that the PRGs exhibit distinct expression changes across cancer types (Figure 2A). However, certain genes displayed consistent expression patterns in the pan-cancer analysis. For example, guanylate binding protein (GBP) family genes were prevalently upregulated, whereas most NLRP family genes were generally downregulated. In contrast, several PRGs, such as *TLR4* and *PYDC1*, showed miscellaneous cancer-type-specific patterns that have not been previously characterized. To dissect the overall changes in the PRGs in different cancers, we calculated the weighted scores of the expression changes. On the basis of this score, 31 cancer types were divided into three categories: the first category was the “high-score group” (>1), of which PRGs were upregulated overall, and which include CESC, CHOL, GBM, HNSC, KIRC, LAML, LGG, OV, PAAD, SARC, STAD and TGCT (marked in red in Figure 2A); the second category was the “low-score group” (<−1), which includes ACC, KICH, LUAD, PRAD, SKCM and THYM (marked in blue in Figure 2A); and the third category was the “middle-score group”, of which the expression of the PRGs did not change significantly (between -1 and 1), and which include BLCA, BRCA, COAD, DLBC, ESCA, KIRP, LIHC, PCPG, READ, THCA, UCEC, and UCS (marked in black in Figure 2A). The bars on the right side show the sum of the multiples of each gene in the different cancer types. Among these 31 types of cancer, there are 22 types with multiple scores greater than 0, which means that the PRGs are increased in these cancers. On the other hand, there are only 9 types of multiples less than 0, which means that the PRGs are generally decreased in these cancers. The comparison demonstrates that PRGs are more likely to be upregulated in cancer. Then, the survival curves were further analyzed for the above three categories (Figure 2B–D). For the first category, named the “high-score group”, the higher the expression of PRGs, the longer the survival time of the patients (Figure 2B). For the second category, named the “low-score group”, the lower the expression of PRGs, the longer the survival time of the patients (Figure 2C). For the third category, named the “middle-score group”, there was no obvious relationship between the expression of PRGs and the survival time (Figure 2D). Therefore, pyroptosis may play distinct roles across different cancer types, and individualized therapeutic strategies may be required in different cancer patients. The survival analysis for each cancer was consistent with the merge group in Figure 2B–D, respectively (Figures S1–S3).

### 3.3. Pan-Cancer Analysis of the Transcriptional Regulation of PRGs

The expression of PRGs was prevalently dysregulated across 31 cancer types (Figure 2A). An examination of the transcriptional regulation of the genomic elements on the genes will help us to better understand the causes of the changes in the expression of PRGs. Copy number variations (CNVs) are important potential sources of human phenotypic variation, and studies have shown that many human diseases with complex traits, such as DiGeorge syndrome [44] and Angelman syndrome [45], are closely related to the copy number variation. MicroRNAs (miRNAs) are major players in gene expression regulation [46]. Enhancers, a type of noncoding DNA element, play critical roles in cancer progression, survival, and treatment outcome [47]. Enhancers are recruited by transcription factors to activate the transcription of target genes through either cis- or trans-interactions [39]. The activation of oncogenes or the oncogenic signaling pathways often converge at the enhancer activation [48]. To delineate the underlying regulatory mechanisms, we collected copy number segments and corresponding gene expression data. The miRNA structure information from ENCORI (http://starbase.sysu.edu.cn/, accessed on 1 May 2021) was used to predict the potential target genes, and the eRNA expression data were from eRic (https://hanlab.uth.edu/eRic/, accessed on 9 October 2021). Combined with the TCGA RNA-Seq data, the expression correlations between miRNAs/enhancers and their target genes were calculated.

**Figure 3:**
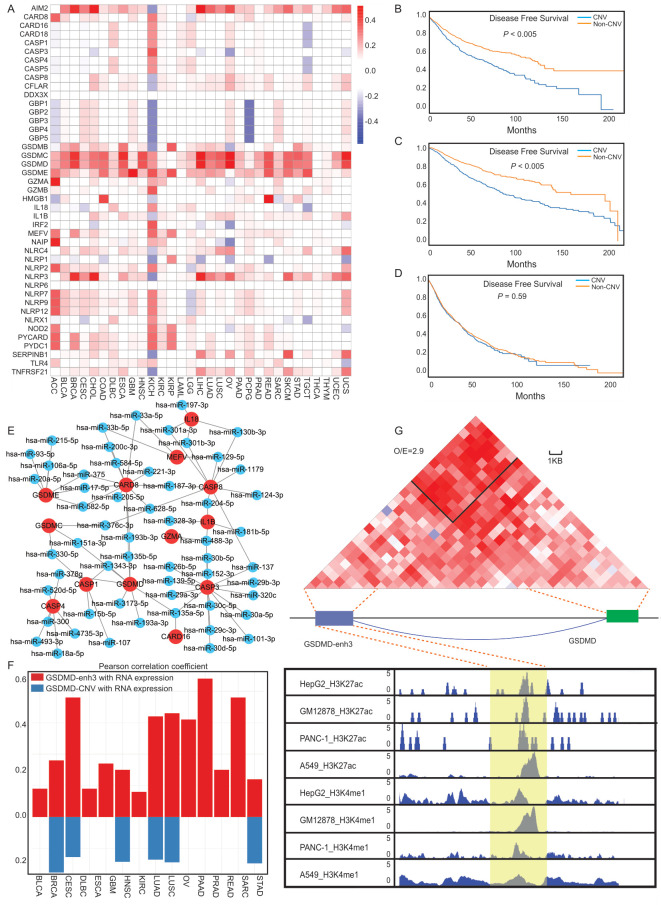
Genome elements on transcriptional regulation of PRGs. (**A**) The heat map showing the CNV changes in PRGs in each cancer type. (**B**–**D**) Kaplan-Meier curves for the CNVs of samples in the high-score (**B**), low-score (**C**), and middle-score (**D**) groups. (**E**) The miRNA-mRNA network for PRGs: the red circles are genes, and the blue circles are miRNAs (Pearson correlation > 0.4, FDR < 0.05). (**F**) The upper red histogram shows the correlation between the enhancer (GSDMD-enh3) and GSDMD gene expression, and the lower blue histogram shows the correlation between CNV and GSDMD gene expression. The correlation statistics adopt the Pearson correlation coefficient statistical method. (**G**) Hi-C results demonstrating the combination of GSDMD and the GSDMD-enh3 at the chromosomal level (upper panel). ChIP-Seq signals of active enhancers (H3K27ac and H3K4me1) in different cancer cell lines (HepG2, GM12878, PANC-1, and A549) (lower panel).

Next, the Pearson correlation between the gene expression and the copy number from the masked copy number segment of the TCGA was examined. First, we obtained the “Segment_Mean” CNVs of 43 PRGs in each cancer type from TCGA (Figure 3A). The “Segment_Mean” value is defined as log_2_(copy_number/2). A value greater than 0 indicates a gain, while a value less than 0 indicates a loss [49]. This value can be used to measure changes in the CNV. We found that the CNV in most PRGs is gained in cancer, such as *GSDMC*, *AIM2*, and *GSDMD*. The CNV changes in KICH cancer were the most intense. Further analysis of the frequency and gene expression alterations within CNV regions (Figure S1A) revealed that, in most cancers, the increase or decrease in gene expression is positively correlated with the amplification or deletion of the CNV regions. In addition, we also analyzed the relationship between the CNV and changes in the survival time. We used GISTIC2 (Version 2.0.23) to set the estimated CNV expression value threshold to −2, −1, 0, 1, and 2, representing homozygous deletion, single copy deletion, diploid normal copy, low-level copy number amplification, and high-level copy number amplification, respectively [50]. Among the 43 PRGs, as long as the copy number change of any gene is not equal to 0, it will be classified into the “CNV group”. The other individuals were assigned to the “non-CNV group”. Analyzing the survival time of the different groups of subjects, we found different patterns of survival curves among the three groups defined in Figure 2A (high-score group, low-score group, and middle-score group). For both the low-score and high-score groups, the CNV significantly reduced the survival times of patients; in contrast, in the middle-score group, the CNV did not significantly change the survival time (Figure 3B–D).

miRNAs are a type of posttranscriptional regulatory factor that downregulate the expression of target genes by acting on mRNAs [46]. To determine which miRNAs are involved in the regulation of pyroptosis, the starBase database was used to establish a miRNA-gene network based on crosslinking-immunoprecipitation and high-throughput sequencing (CLIP-Seq) data and degradation data. Then, the potential miRNA target genes were inferred, and those miRNAs that were significantly correlated with the expression of PRGs were proposed to be involved in the regulation of pyroptosis [51]. The Pearson’s correlation between the expression of the miRNAs and the PRGs was statistically evaluated (Pearson correlation > 0.4, FDR < 0.05). Cytoscape software (Version 2.8.2) was used to visualize the high-frequency interaction networks among the PRGs and miRNAs [52]. As the network shows (Figure S3F), all interactions between the miRNAs and PRGs occurred in more than 10 cancer types. PRGs, such as *GSDME*, *GSDMD*, and *CASP3*, are potential targets of multiple miRNAs, demonstrating that the expression of PRGs is tightly regulated.

We not only analyzed the effects of CNV and miRNA on gene expression, but we also characterized the enhancers regulating PRGs. The expression levels of eRNA (enhancer RNA) are generally used as an index for enhancer activity. Therefore, we first combined the eRNA location information from the eRic database and determined eight different enhancers near the six PRGs (*GZMA*, *CASP3*, *CASP8*, *GSDMA*, *GSDMB*, and *GSDMD*) within 1 Mb [39]. For *GSDMD*, three enhancers (GSDMD-enh1, GSDMD-enh2, and GSDMD-enh3) were predicted to regulate the expression of *GSDMD*. By examining the Pearson correlation between PRGs and its possible enhancers (Figure S1B), we found that genes and enhancers with higher correlations only appeared in a subset of cancer types, not in all the cancer types. This is most likely due to the specificity of the enhancers. The correlation between the CNV and RNA expression changes demonstrated that the change in the gene expression was not caused by chromosomal alterations (Figure S1A). Among the enhancers corresponding to these genes, GSDMD-enh3 had the strongest correlation with *GSDMD* gene expression. In 16 types of cancer, the correlation between *GSDMD*-enh3 and *GSDMD* expression was greater than that between CNV and *GSDMD* expression, suggesting that the alteration of the *GSDMD* gene expression is mainly caused by the activity change in the GSDMD-enh3 (Figure 3F).

H3K27ac and H3K4me1 are canonical markers of active enhancers. There are high binding peaks around the GSDMD-enh3 on the ChIP-Seq data in various cancer cell lines (HepG2, GM12878, PANC-1, and A549), indicating that there are potential enhancers upstream of *GSDMD* (Figure 3G). High-throughput chromosome conformation capture (Hi-C) data analysis validated the association between the GSDMD-enh3 and *GSDMD* at the chromosomal level (Figure 3G). In the HACER database (http://bioinfo.vanderbilt.edu/AE/HACER/index.html, accessed on 9 October 2021), GSDMD-enh3 is predicted to combine with *GSDMD* through the USF1(Upstream Transcription Factor 1) transcription factor. ChIP-Seq data of USF1, available for the K562, HCT-116, and H1 cell lines thus far, demonstrated that USF1 can bind to both *GSDMD* and the GSDMD-enh3 (Figure S1C). On the basis of the analysis above, we predict that the GSDMD-enh3 regulates the expression of *GSDMD* through USF1 to induce pyroptosis.

### 3.4. Construction of a Cancer Prognostic Model in the TCGA Cohort Based on the Expression of PRGs

**Figure 4:**
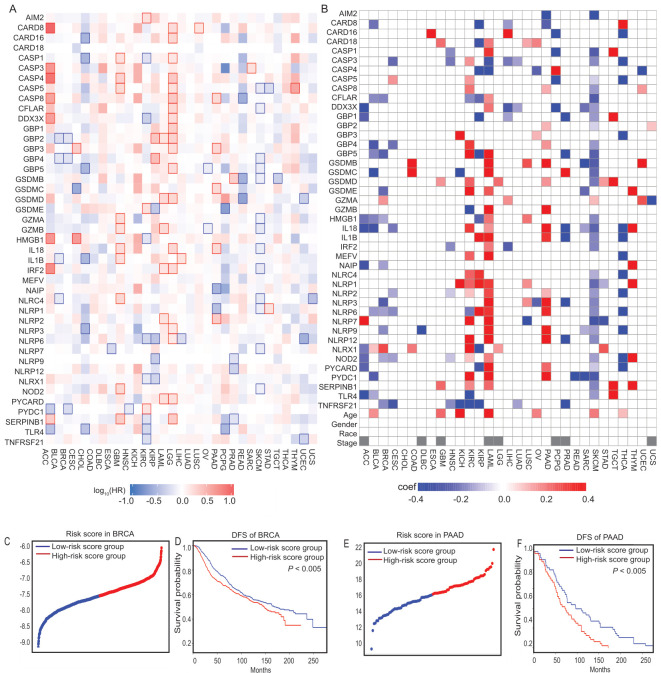
The prognostic values of PRGs. (**A**) The survival contribution between 43 PRGs and DFS. The heat map shows the hazard ratios on a logarithmic scale (log10) for different genes. The red and blue blocks denote higher and lower risks, respectively. The rectangles with frames indicate significantly unfavorable and favorable results in prognostic analyses. (**B**) Risk model across 31 cancer types. Because of the limited sample size for clinical cancer stage information, 8 types of cancer were excluded from the analysis (marked as gray box). (**C**) The distribution of the risk scores for BRCA from ICGC data. (**D**) Kaplan-Meier curves for the DFS of patients in the high-risk and low-risk groups for BRCA from ICGC data. (**E**) The distribution of the risk scores for PAAD from ICGC data. (**F**) Kaplan-Meier curves for the DFS of patients in the high-risk and low-risk groups for PAAD from ICGC data.

Most PRGs were differentially expressed between cancer and noncancerous cells (Figure 2A), and they were correlated with DFS in the survival analysis (Figure 4A). Considering that demographic information (age, gender, race, cancer stage) may also affect patient survival, in order to better simulate the patient survival probability, a regression method that can process all independent variables at the same time is required [53]. Therefore, LASSO Cox regression analysis was applied to establish a prognostic model using the expression profile of the 43 genes mentioned in Table S1 and the four demographic factors above. Because of limited cancer stage data, we only included this factor in 23 cancer types. For each cancer type, first, we used the Schoenfeld residual method to judge the proportional hazards assumption of the Cox proportional hazard regression analysis, which demonstrated that the risk ratio does not change with time, and that each covariate satisfied the risk ratio assumption (Figure S5). Then, the degree of the LASSO regression complexity adjustment was determined using the parameter, λ. On the basis of the best λ value, we built the risk model for each cancer type (Figure 4B). The greater the deviation of the coefficient from 1, the greater the impact on the risk score. Patients were stratified into different groups by the median cutoff values of the risk scores within each cancer type: those above the median were the high-risk group, while those below the median were the low-risk group.

Survival analyses were performed on different cancer risk models to test the correctness of the models (Figures S6–S8). For CHOL, since all of the factors’ effect is not significant (*p* > 0.05), it failed to build the risk model for this cancer type. Because of the small sample size (sample number < 60), we didn’t perform survival analyses by groups for DLBC and UCS. For the remaining 28 cancer types, the patients in the low-risk group had a longer survival probability in 26 cancer types. To test the robustness of the model constructed from TCGA patients, the International Cancer Genome Consortium (ICGC) data of BRCA and PAAD were also categorized into high- or low-risk groups by the median value calculated using the same formula as that for TCGA patients. Similar to the TCGA cohort, patients in the high-risk group survived significantly shorter than those in the low-risk group (Figure 4C–F). These results indicate that the risk survival model has good applicability, and that the risk score is optimal as a prognostic indicator of DFS.

### 3.5. Exploring the Prognostic Significance of PRGs in Immunity on the Basis of the Risk Model

**Figure 5:**
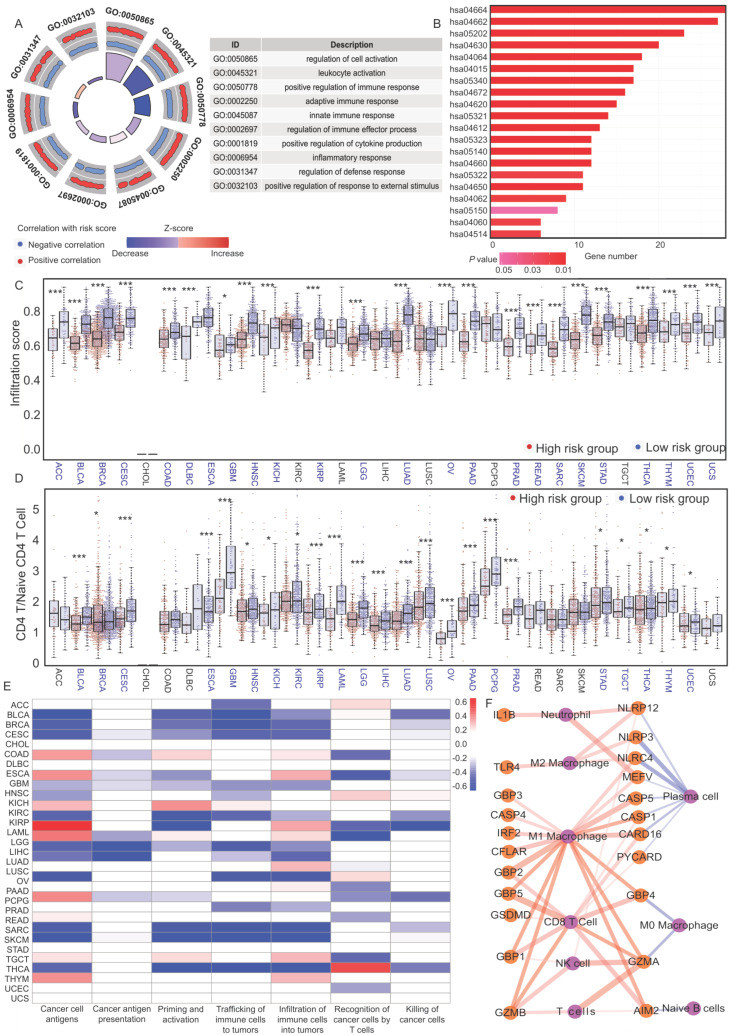
Functional analysis of the risk-correlated genes. (**A**) For the risk-score-related genes, the most enriched GO terms. The outermost circle is the correlation between the gene expression and the risk score. The height of the middle bar graph shows the number of genes enriched in this pathway, and the color is the Z-score. (**B**) For the risk-score-related genes, the most enriched KEGG terms. Among the 31 cancer types of TCGA, the difference in the infiltration score (**C**) and the ratio of CD4 T cells and naive CD4 T cells in low and high groups stratified by the pyroptosis risk-score (**D**) * denotes *p* < 0.05 and *** denotes *p* < 0.001. (**E**) The correlation between risk scores and immunization processes. Red denotes a positive correlation between the immune activity score and the risk score, while blue denotes a negative correlation (Pearson’s correlation > 0.4, *p* < 0.05). (**F**) The PRG immune cell network. The orange circles represent genes, while the blue circles represent immune cells. The red lines represent a positive correlation (Pearson’s correlation > 0.4, *p* < 0.05, no. of cancer types > 25), while the blue lines represent a negative correlation. The thickness of the lines represents the number of cancer types. “T cells” stands for Gamma Delta T cells.

Although there are an increasing number of studies on cell pyroptosis, the landscape has not been sufficiently explored. In order to identify the potential genes involved in the process of pyroptosis, the correlation between the expression of all genes and the risk scores in different cancers was established using a risk model. Since the risk score in this study is calculated on the basis of the expression changes of the PRGs, we propose that if a gene has the same trend in the Pearson correlation as the risk scores in multiple cancers (n > 5), then the gene is potentially associated with pyroptotic processes. From the Pearson correlation coefficient, we divided these genes into two categories. The genes in the first category had a positive correlation with the risk scores in at least five cancer types, and no negative correlation with other cancer types (Table S4). On the other hand, the genes in the second category exhibited a negative correlation with the risk scores in at least five cancer types, and no positive correlation in the other cancer types (Table S5). GO (gene ontology) and KEGG enrichment analyses for these genes were performed, which demonstrated that their functions were primarily enriched in the immune response, inflammation, and cytokine enrichment (Figure 5A,B).

Considering the important role in the immune process, we then assessed the immune infiltration scores and the ratios between the different immune cell types across 31 cancer types in the high-risk group and the low-risk group, respectively. Except for the three types of cancers (KIRC, LUSC, TGCT), the degree of the infiltration scores of patients in the low-risk group was not lower than that in the high-risk group (Figure 5C). The ratio between CD8 T cells, CD4 T cells, and macrophages were calculated in this study, but we found that only the ratio between CD4 T cells and naive CD4 T cells was negatively correlated with cancer patient survival across different cancer types, except DLBC (Figure 5D). Therefore, the ratio between CD4 T cells and naive CD4 T cells may be applied as an index for immune infiltration.

To further characterize the role of risk scores in cancer immunity, we used ImmuCellAI (Immune Cell Abundance Identifier) (http://bioinfo.life.hust.edu.cn/ImmuCellAI#!/analysis, accessed on 22 December 2021) and the TIP (tracking tumor immunophenotype) (http://biocc.hrbmu.edu.cn/TIP/index.jsp, accessed on 22 December 2021) to quantify the enrichment score of the degree of tumor immune invasion and the cancer-immunity cycle, respectively [54,55]. The cancer-immunity cycle is divided into the following seven steps: the release of cancer cell antigens (Step 1); cancer antigen presentation (Step 2); priming and activation (Step 3); the trafficking of immune cells to tumors (Step 4); the infiltration of immune cells into tumors (Step 5); the recognition of cancer cells by T cells (Step 6); and the killing of cancer cells (Step 7) [55]. Through the measurements of the different steps and the Cox risk scores, we found that, in most cancers, pyroptosis affects the trafficking of immune cells to tumors (Figure 5E, blocks are shown in red or blue). If changes in the expression of PRGs affect the composition of immune cells, it would be of great value to combine pyroptosis perturbations with the current cancer treatments. CIBERSORT (Version 1.03) is currently the most widely used tool to calculate the proportion of immune cells in different samples. The correlation between the expression of PRGs and the proportion of different immune cells was calculated. Surprisingly, the expressions of many PRGs, such as the *GZMA* and GBP family genes, were associated with the proportion of immune cells, and PRGs primarily affected the ratio of macrophages, T cells, and B cells (Figure 5F). The proportion of M0 macrophages was negatively correlated with the expression of *GBP4* and *GZMA* (blue lines), and the proportion of M1/M2 macrophages was positively correlated with the expression of PRGs (red lines). This result indicates that, with the increase in PRG expression, in most cancer patients, M0 macrophages decrease, while both M1 and M2 macrophages increase; the numbers of T cells and NK cells increase, while the proportion of B cells decreases in cancer.

In conclusion, it is more likely that the pyroptosis process affects the cancer patient survival time by changing the proportion of immune cells, which may result in immune microenvironment changes. Therefore, the induction of pyroptosis may influence treatment outcomes. A recent study indicated that pyroptosis may play an important role in the ability of immune cells to attack tumors, as well as in the regulation of the tumor immune microenvironment [56], consistent with our observations. Therefore, pyroptosis may be further explored to augment immunotherapy therapies.

### 3.6. Exploration of PRGs to Predict Immunotherapeutic Outcomes

**Figure 6:**
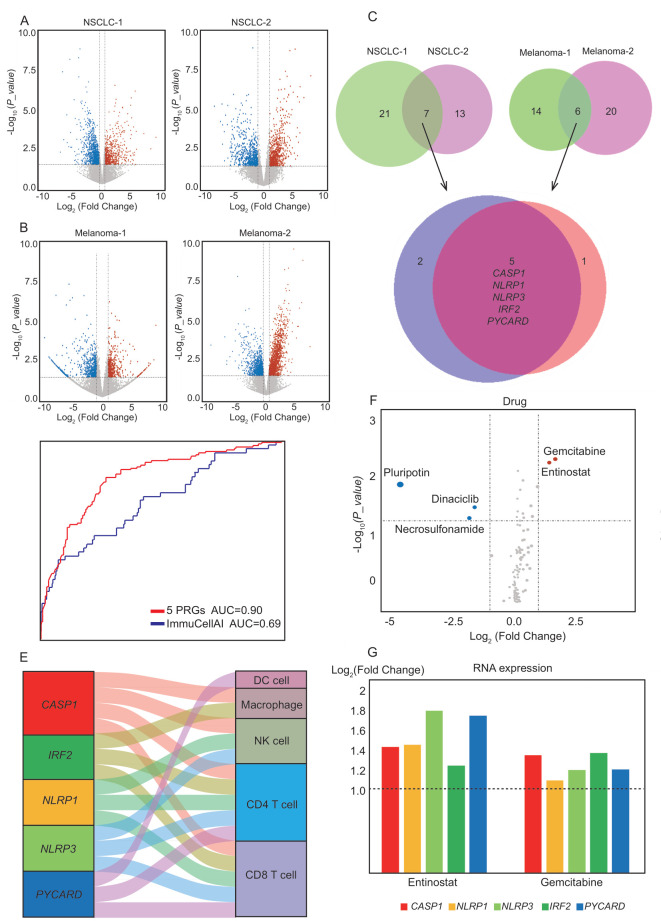
The effects of PRGs on immunotherapy. (**A**,**B**) Volcano plot of differentially expressed genes between responders and nonresponders to pembrolizumab or nivolumab in NSCLC and melanoma. The red dots in the plot represent upregulated genes, while the blue dots represent downregulated genes in responders (fold change > 1.5, *P_adj_* < 0.05). (**C**) Differentially expressed PRGs from NSCLC and melanoma. (**D**) Comparison of predictive ability of 5 PRGs and ImmuCellAI for immunotherapy population using ROC curves analysis. (**E**) Sankey diagram shows the relationship between 5 PRGs and the immune cells (DC cells, macrophage cells, NK cells, CD4+ Treg cells, and CD8+ Treg cells) in cancer patients with immunotherapy (Pearson coefficient > 0.6). (**F**) The volcano map shows the effect of different anticancer chemical drugs on the expression of 5 PRGs. The red dots in the figure represent drugs that upregulate the expression of PRGs, while the blue dots represent the drugs that downregulate the expression of PRGs. (log_2_FoldChange > 1, *P_adj_* < 0.05). (**G**) The bar plot shows changes in PRGs in response to five anticancer drugs. The crimson bar is *CASP1*, the yellow bar is *NLRP1*, the light green bar is *NLRP3*, the green bar is *IRF2*, and the blue bar is *PYCARD*. The vertical axis represents the multiples of the expression changes.

From the above analysis, we found that the expression of PRGs is closely related to the immune process. Next, we determined whether the expression of PRGs could predict immunotherapeutic outcomes. First, we collected the RNA-Seq data of immunotherapy for NSCLC (GSE78220 and GSE115821) and melanoma (GSE135222 and GSE126044) from the GEO database (https://www.ncbi.nlm.nih.gov/geo/, accessed on 8 November 2021). Then, a variance analysis was performed using Deseq2 in these four datasets. Those with an expression fold change > 1.5 and a *p* < 0.05 were defined as differentially expressed genes between patients with and without immunotherapy response (Figure 6A,B). Among the forty-three PRGs, five were highly expressed in the four populations of immunotherapy responders, including *CASP1*, *NLRP1*, *NLRP3*, *PYCARD*, and *IRF2* (Figure 6C). Can these five PRGs be used as biomarkers to effectively classify the cancer patients into different populations with diverse responses to immune therapy? In the four RNA-Seq datasets, the predictive ability of the five PRGs above was significantly greater than ImmuCellAI, which is a current commonly used webpage for predicting the abilities of immune responses (Figure 6D).

By calculating the relationship between the immunocyte composition and the gene expression in immunotherapy responders, the proportions of CD4 T cells, CD8 T cells, DC cells, macrophages, and NK cells were positively correlated with the expression of *CASP1*, *NLRP1*, *NLRP3*, *PYCARD*, and *IRF2*, respectively, in most cancers (Figure 6E). However, this correlation was not significant in cancer patients (Figure 5F), so it may be a characteristic specific to the immunotherapy-responsive population. In other words, in most cancer types, when the expression of these five genes is upregulated, the T-cell infiltration in the tumor tissue will be correspondingly improved, thus effectively promoting immunotherapy.

To improve the outcome of immunotherapy, we collected H-STS drug perturbation datasets 24 h after perturbation with small-molecule compounds (GSE96760) [57]. On the volcano map, drugs corresponding to the red dots effectively promoted the expression of these five genes, while drugs corresponding to the blue dots inhibited the expression of these genes. According to changes in the gene expression, we found that gemcitabine and entinostat effectively promoted the expression of genes, such as *CASP1* (Figure 6E). Necrosulfonamide is an inhibitor of pyroptosis [58], which was also confirmed by the volcano plot results (Figure 6E). To further determine the role of these drugs, we collected RNA-seq data from GSE165548 (gemcitabine) and GSE131085 (entinostat). The expression of the five genes was stimulated by both drugs (Figure 6F). The combination of the pyroptosis promotion by gemcitabine/entinostat and immunotherapeutic drugs may be a potential therapeutic strategy for cancer. Studies have confirmed the stimulating effect of entinostat on immunotherapy, which shows that the results obtained by the analysis here are credible [59,60,61].

## 4. Discussion

Immunotherapy, as a treatment method that stimulates the original ability of the immune system to fight tumor cells, is a current research hotspot [62,63]. An increasing number of studies have found that pyroptosis plays an essential role in the immune process. Pyroptosis can inhibit the growth of cancer cells and stimulate inflammation, representing a potential new strategy for cancer immunotherapy [37]. However, only a limited number of genes have been identified as tumor suppressors [64,65]. In this study, we determined the function of pyroptosis and its application value in immunotherapy from different perspectives. We found that the lack of gene pathway integration is one of the difficulties in the current research, which has led to an inconsistent understanding of the process of pyroptosis by different researchers and has caused current research to only focus on the functions of a few genes. For the first time, we generated a list of 43 genes involved in the process of pyroptosis, including genes that perform pyroptosis, regulate the expression of the pyroptotic core genes, and encode inflammatory factors. The generalization of PRGs will be of great help for examining the functions of PRGs from a biological perspective.

Studies have shown that *GSDMC* may act as an oncogene to promote the occurrence of colorectal and lung adenocarcinoma [66], while *GSDME* acts as a tumor suppressor in breast and liver cancer [67]. The results of our omics study are consistent with previous findings, and we discovered the opposite effect of some genes in other cancers. The inconsistent performance of PRGs in different cancers hints that pyroptosis is temporospatially specific. However, our gene set does not exclude the influence of other pathways. Among the genes we have selected, certain genes are involved in other types of programmed cell death, such as apoptosis. In fact, it is impossible to clearly determine that one gene is only involved in pyroptosis and not apoptosis. For example, besides pyroptosis, *CASP3* and *NLRP3* inflammasomes are also involved in apoptosis [68,69]. This is one of the limitations of this study. However, just like the selection of other genetic pathways, we cannot avoid this problem.

The statistical analysis of the overall changes in PRGs between different cancers showed these changes from the perspective of pan-cancer, guiding future research. Nevertheless, one limitation is that this research failed to clarify the reason for the changes in the pyroptosis gene expression among different cancers; the current classification of different cancers is too simple to analyze from the viewpoint of the biological characteristics of cancers.

At the DNA level, we characterized the potential enhancers regulating *GSDMD* on the basis of ChIP-Seq, and the Hi-C. GSDMD-enh3 exhibits relatively high binding peaks in a variety of cancer cell lines, indicating that targeting the GSDMD-enh3 is more likely to be applicable for a variety of cancers. However, it is necessary to perform a functional analysis of the relationship between the GSDMD-enh3 and GSDMD in order to determine the underlying relationship between the GSDMD-enh3 and cancer.

It should be noted that these analyses are mainly based on RNA-seq data from TCGA. However, the regulation of pyroptosis is multilayered. Besides the regulation by the CNV, miRNA, and enhancers that we explored in this study, epigenetic modifications and post-translational modifications also play important roles in pyroptosis in cancer. Since the conclusions of this study were drawn from a bioinformatic analysis, the function of the PRGs should be further validated and depicted.

Although some previous studies have indicated that several PRGs may play entirely distinct roles in different cancer types, the correlation between the expression change of pyroptotic genes and DFS in cancer patients remains largely unknown. As we expected, PRGs were differentially expressed between tumor and normal cancer data, and, in the univariate Cox regression analysis, more than half of them were related to DFS (Figure 4A). These results indicate the potentially important role of pyroptosis in cancer, as well as the possibility of using these PRGs to establish a prognostic model. On the basis of the risk scores between different cancers, we conducted a GO analysis and unexpectedly found that many immune and inflammation-related biological processes and pathways have been enriched.

Pyroptosis may affect the immune cell microenvironment, to a certain extent, by changing the proportion of immune cells, which determine the outcome of immunotherapy [70,71]. Interestingly, certain genes were positively correlated with the risk scores in most cancers, while other genes were negatively correlated with the risk scores (Figure 4B). This divergence may be caused by differences in the release and delivery efficiency of inflammatory factors in different cancer microenvironments. In addition, we analyzed the correlation between the risk score and the process of immune infiltration. The pyroptotic process changes the proportion of immune cells. At present, there are limited studies on pyroptosis and immune infiltration. For example, one study indicated that *GSDME* induces T-cell activation by promoting the infiltration of the T cells into tumors (19). Although many PRGs play a role in the immune process, how the process of pyroptosis stimulates changes in the immune system is one direction for future research.

Can pyroptosis be leveraged for immunotherapy? In this study, we found that five PRGs were dramatically upregulated in patients who responded to immunotherapy (Figure 6C). However, this relationship has not been further experimentally verified, and it is unclear whether the observed changes in the immune cells are caused by the expression changes in PRGs. Although we identified several possible drugs, further research on drugs is needed. From these research results, it can be concluded that the effective overexpression of specific PRGs is beneficial for the response to immunotherapy, and further verification of this process will be one of our future studies.

## 5. Conclusions

We curated and compiled PRGs and defined a risk prediction model for 31 cancer types. This model was further confirmed in the validation cohort, providing a method for cancer risk diagnosis. The underlying mechanism between PRGs and tumor immunity is still poorly understood and warrants further investigation. These results emphasize the important role of the pyroptotic process in cancer immunity and are helpful for further studying the underlying molecular mechanisms, and for developing individualized treatments on the basis of the expression of PRGs. Studies on pyroptosis and cancer are intriguing and represent an exciting field for improving the therapeutic outcomes to overcome cancer.

## Data Availability

The datasets used and/or analyzed during the current study are available from the corresponding author upon reasonable request.

## Supplementary Materials

The following supporting information can be downloaded at: https://www.mdpi.com/article/10.3390/cancers14010237/s1: Figure S1: Kaplan-Meier curves for the disease-free survival (DFS) of samples in the high-score group of Figure 2B. Figure S2: Kaplan-Meier curves for the disease-free survival (DFS) of samples in the low-score group cancer of Figure 2C. Figure S3: Kaplan-Meier curves for the disease-free survival (DFS) of samples in the low-score group cancer of Figure 2D. Figure S4: Regulation of PRGs by copy number variation (CNV) and enhancers. Figure S5: Proportional hazard hypothesis test; Figure S6. The LASSO Cox variable screening; Figure S7. Kaplan-Meier curves for the DFS of patients in the high-risk and low-risk groups for each cancer type (ACC, BLCA, BRCA, CESC, COAD, ESCA, GBM, HNSC, KICH, and KIRC); Figure S8. Kaplan-Meier curves for the DFS of patients in the high-risk and low-risk groups for each cancer type (LGG, LIHC, LUAD, LUSC, OV, PAAD, PCPG, and PRAD). Figure S9: Kaplan-Meier curves for the DFS of patients in the high-risk and low-risk groups for each cancer type (READ, SARC, SKCM, STAD, TGCT, THCA, THYM, and UCEC). Table S1: Functional classification of genes involved in pyroptosis. Table S2: The abbreviations, classification and numbers of samples for the 31 cancer types investigated in this study; Table S3: The function description of KEGG pathway IDs. Table S4: Pearson correlations of genes that are positively correlated with risk scores. Table S5: Pearson correlations of genes that are negatively correlated with risk scores.
