# Supplementary material for: Comprehensive Pan-Cancer Analyses of Pyroptosis-Related Genes to Predict Survival and Immunotherapeutic Outcome
Source: Cancers (Basel). 2022 Jan 4;14(1):237. doi: 10.3390/cancers14010237 (PMC8750048; doi:10.3390/cancers14010237)
Supplement: Supplementary file 1 [file cancers-14-00237-s001.zip › cancers-1491905-supplementary/Supplementary Files/Supplemental Information.pdf]

Figure S1. Kaplan-Meier curves for the disease-free survival (DFS) of samples in the high-score group of Figure 2B.

Figure S2. Kaplan-Meier curves for the disease-free survival (DFS) of samples in the low-score group cancer of Figure 2C.

Figure S3. Kaplan-Meier curves for the disease-free survival (DFS) of samples in the middle-score group cancer of Figure 2D.

Figure S4. Regulation of PRGs by copy number variation (CNV) and enhancers.

Figure S5. Proportional hazard hypothesis test.

Figure S6. The LASSO Cox variable screening.

Figure S7. Kaplan-Meier curves for the DFS of patients in the high-risk and low-risk groups for each cancer type (ACC, BLCA, BRCA, CESC, COAD, ESCA, GBM, HNSC, KICH, and KIRC).

Figure S8. Kaplan-Meier curves for the DFS of patients in the high-risk and low-risk groups for each cancer type (LGG, LIHC, LUAD, LUSC, OV, PAAD, PCPG, and PRAD).

Figure S9. Kaplan-Meier curves for the DFS of patients in the high-risk and low-risk groups for each cancer type (READ, SARC, SKCM, STAD, TGCT, THCA, THYM, and UCEC).

Tables S1. Functional classification of genes involved in pyroptosis.

Tables S2. The abbreviations, classification and numbers of samples for the 31 cancer types investigated in this study.

Tables S3. The function description of KEGG pathway IDs.

Tables S4. Pearson correlations of genes that are positively correlated with risk scores.

Tables S5. Pearson correlations of genes that are negatively correlated with risk scores.

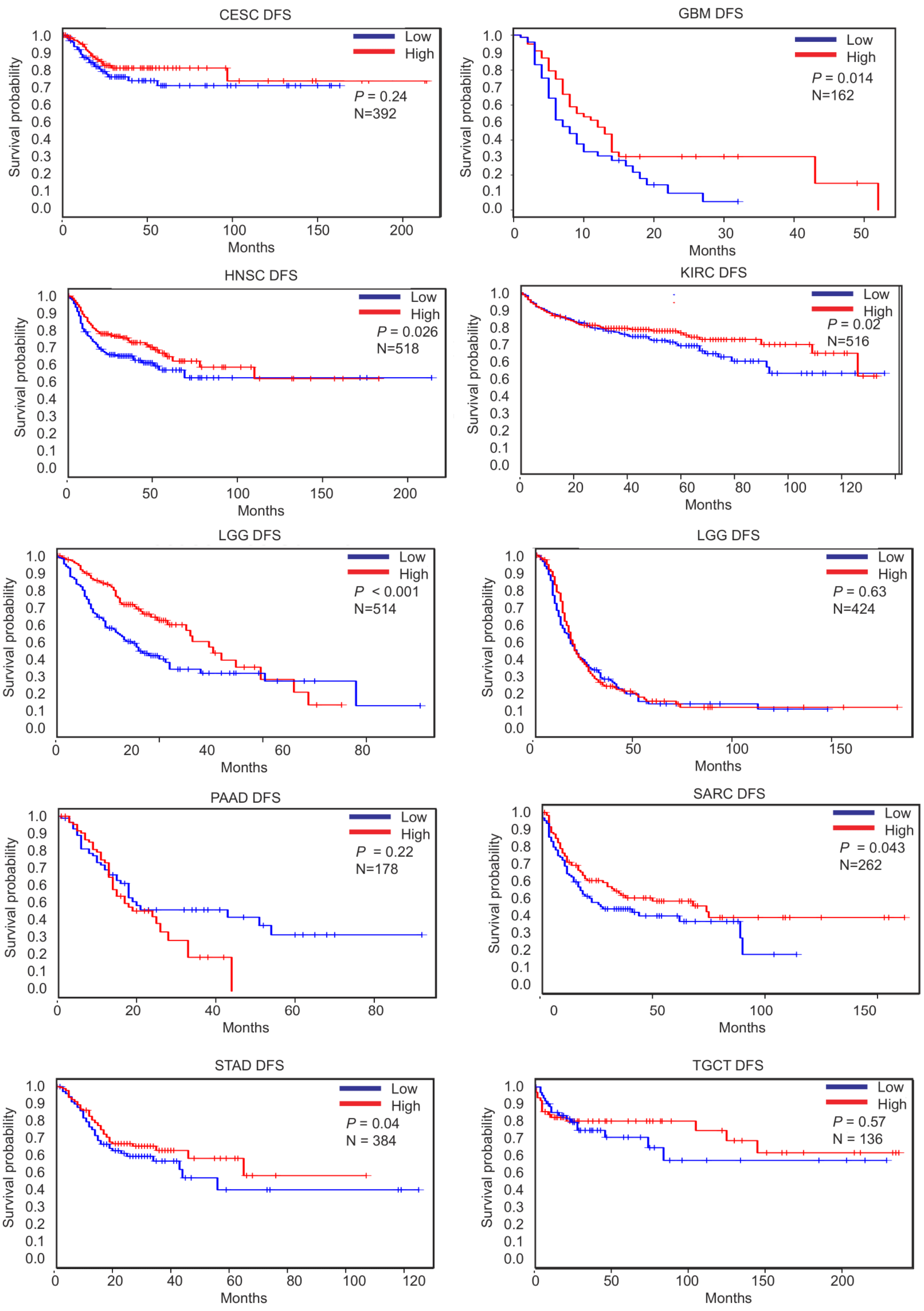

Figure S1 Kaplan-Meier curves for the disease-free survival (DFS) of samples in the high-score group of Figure 2B.

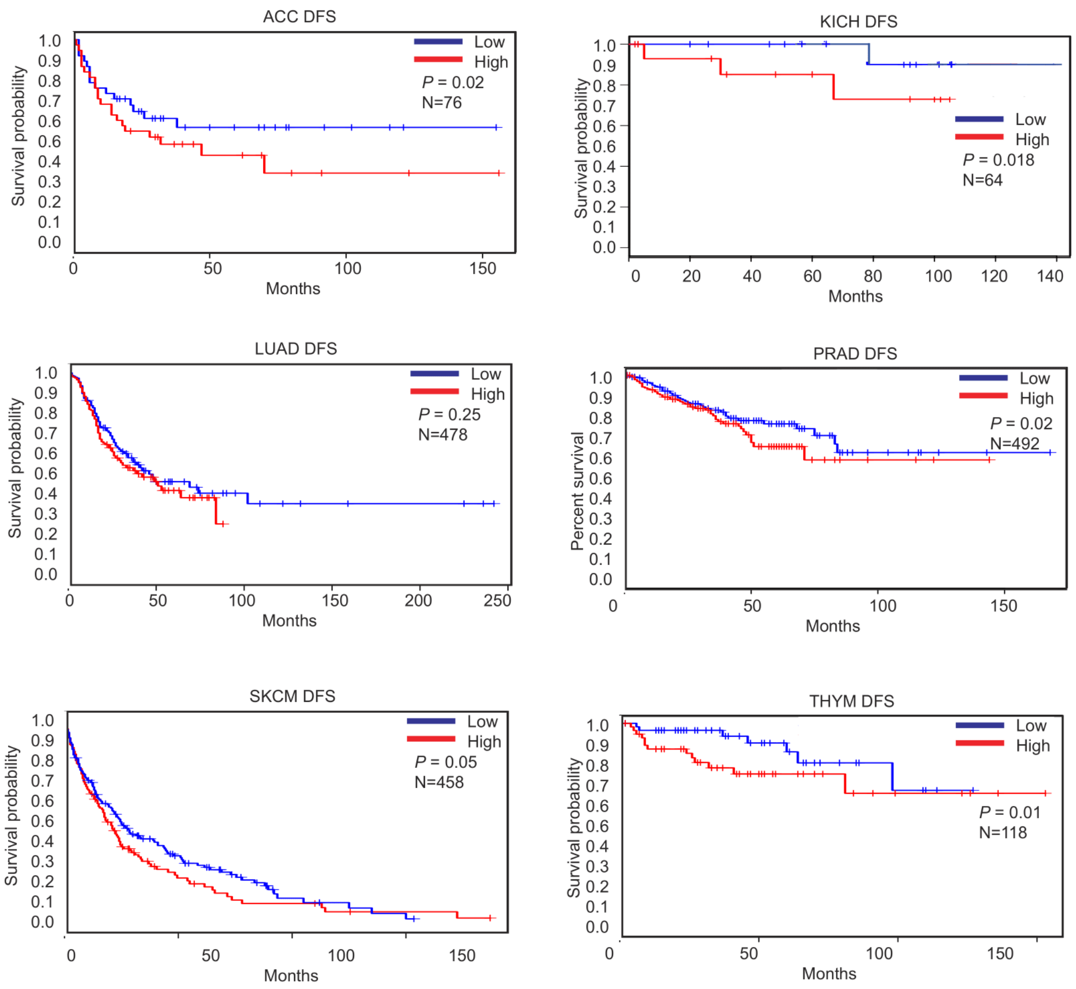

Figure S2 Kaplan-Meier curves for the disease-free survival (DFS) of samples in the low-score group of Figure 2C.

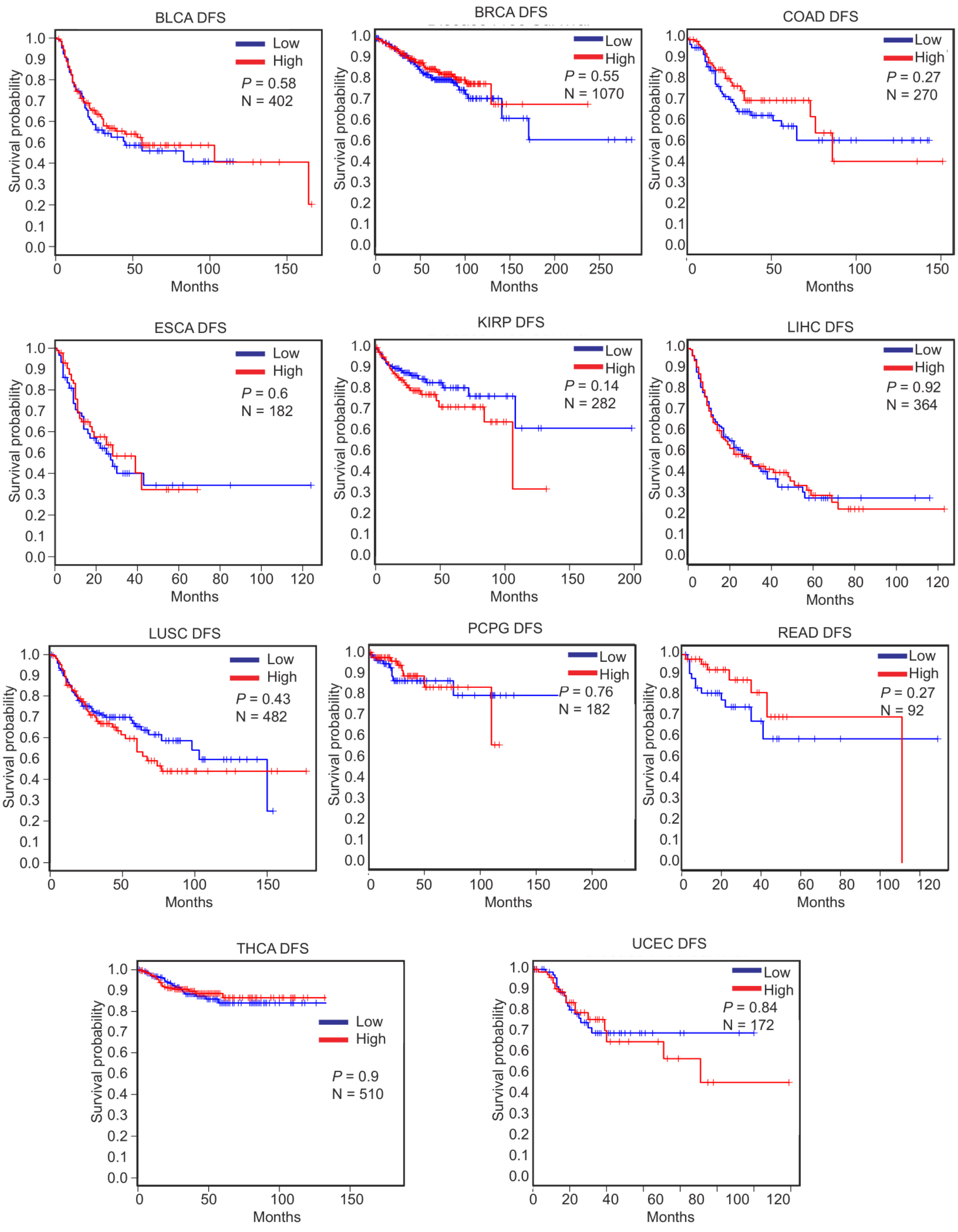

Figure S3 Kaplan-Meier curves for the disease-free survival (DFS) of samples in the middle-score group of Figure 2D.

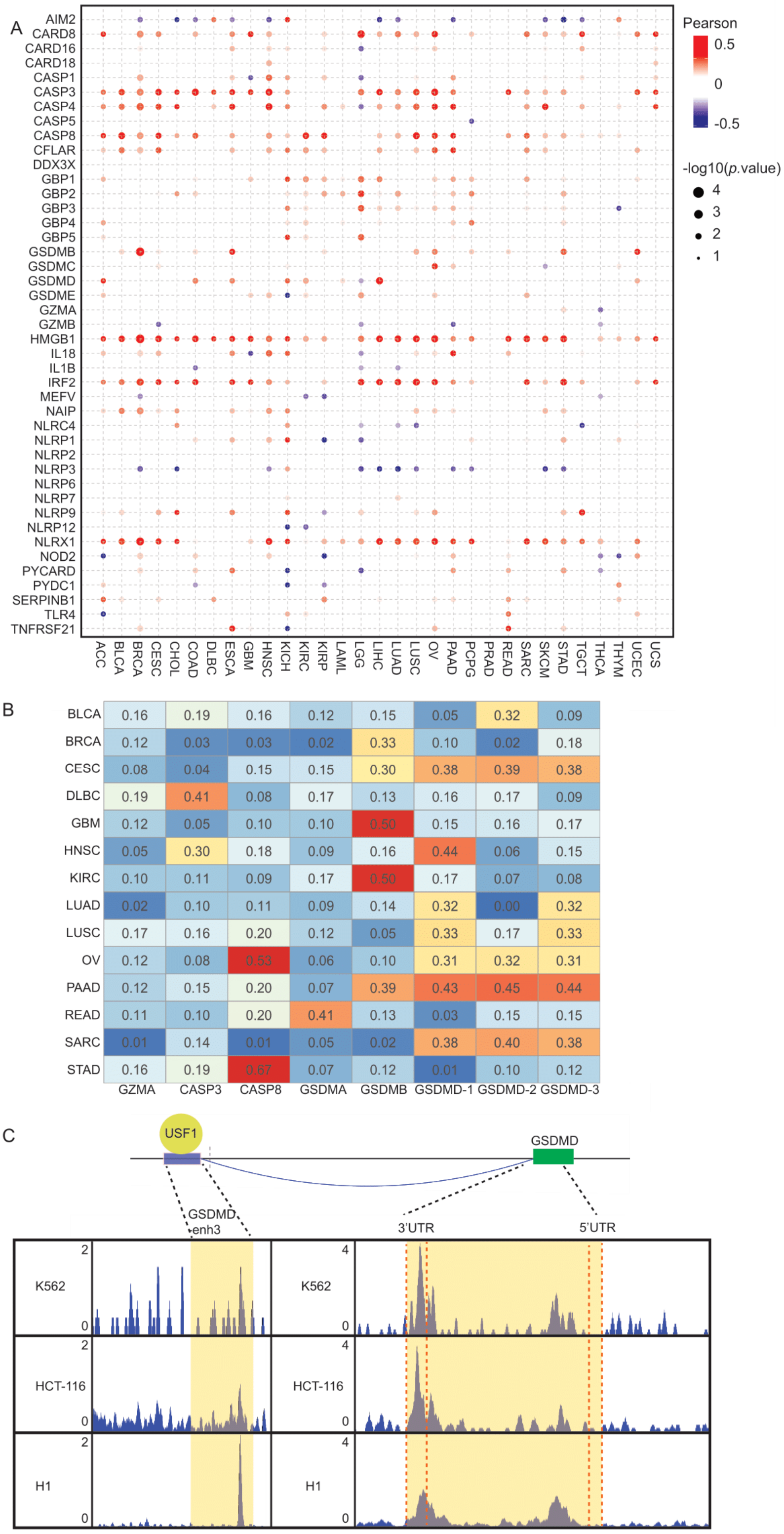

Figure S4 Regulation of PRGs by copy number variation (CNV) and enhancers. (A) Pearson's correlation between CNV and the expression of PRGs. (B) The expression correlation between PRGs and potential enhancers. (C) ChIP-Seq signals of

transcription factor USF1 demonstrates that USF1 may bind to both GSDMD-enh3 and *GSDMD* simultaneously in cell lines K563, HCT-116 and H1, respectively.

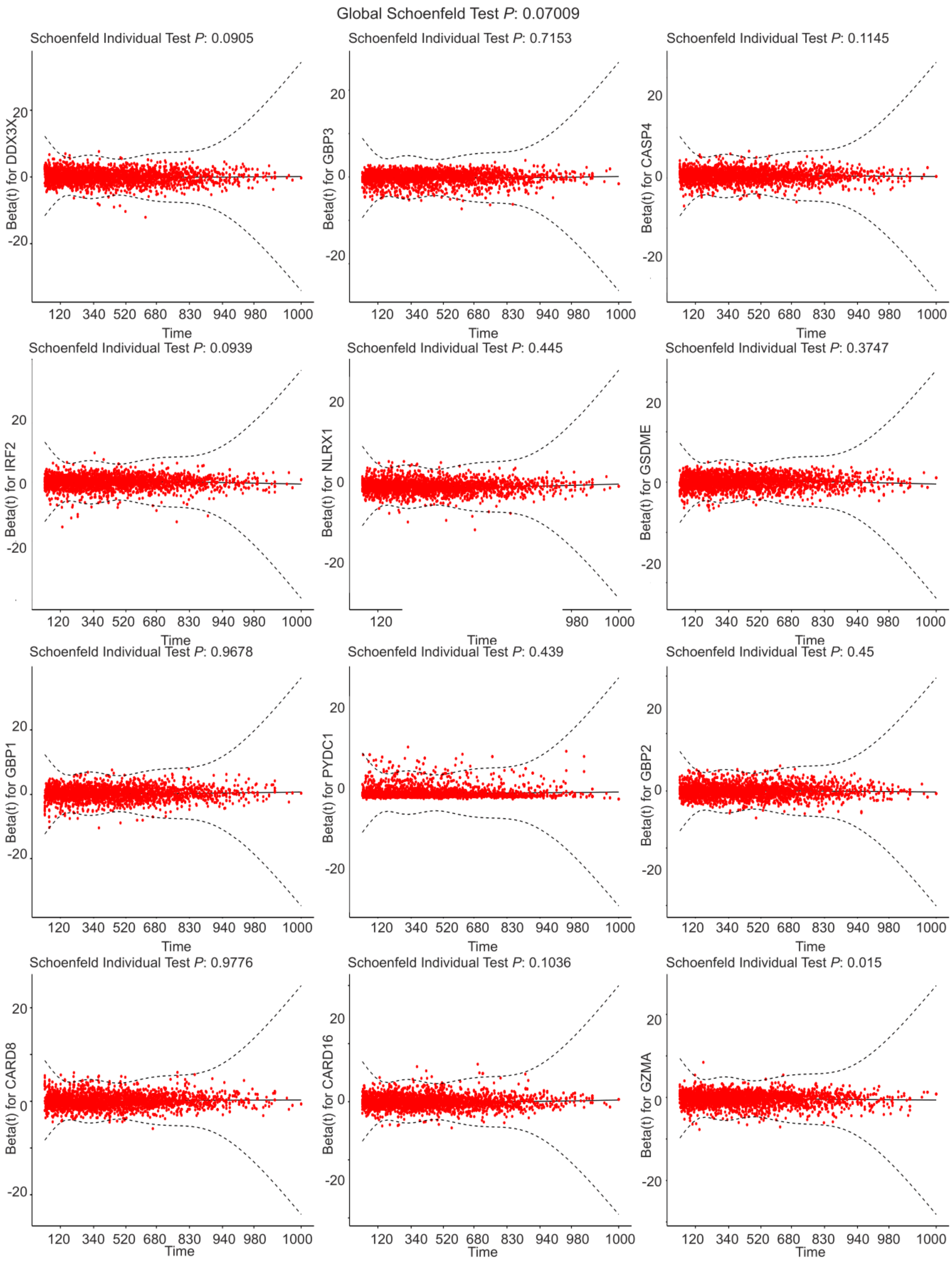

Figure S5 Proportional hazard hypothesis test. Schoenfeld residual method judges the Proportional Hazards assumption of Cox proportional hazard regression analysis. The solid line is a smooth spline curve fitted to the curve, and the dashed line represents the +/- 2 standard error band around the fitting.

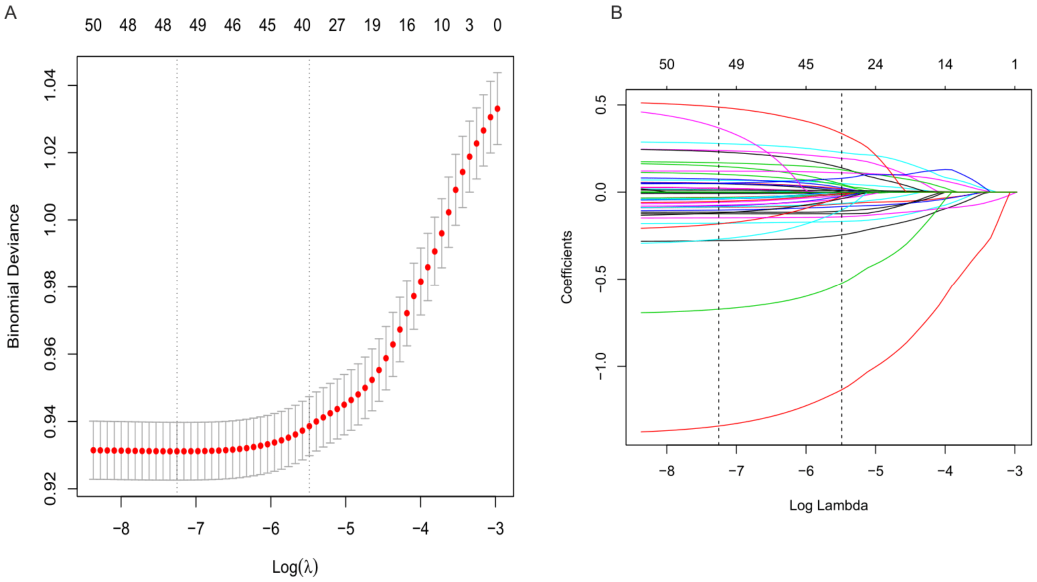

Figure S6 The LASSO Cox variable screening. (**A**) The LASSO coefficient profiles of PRGs. LASSO, least absolute shrinkage and selection operator. (**B**) Ten-time cross-validation for tuning parameter selection in the LASSO model. Each curve represents the change trajectory of each gene, the ordinate is the coefficient value, and the upper abscissa is the number of non-zero coefficients in the model at this time.

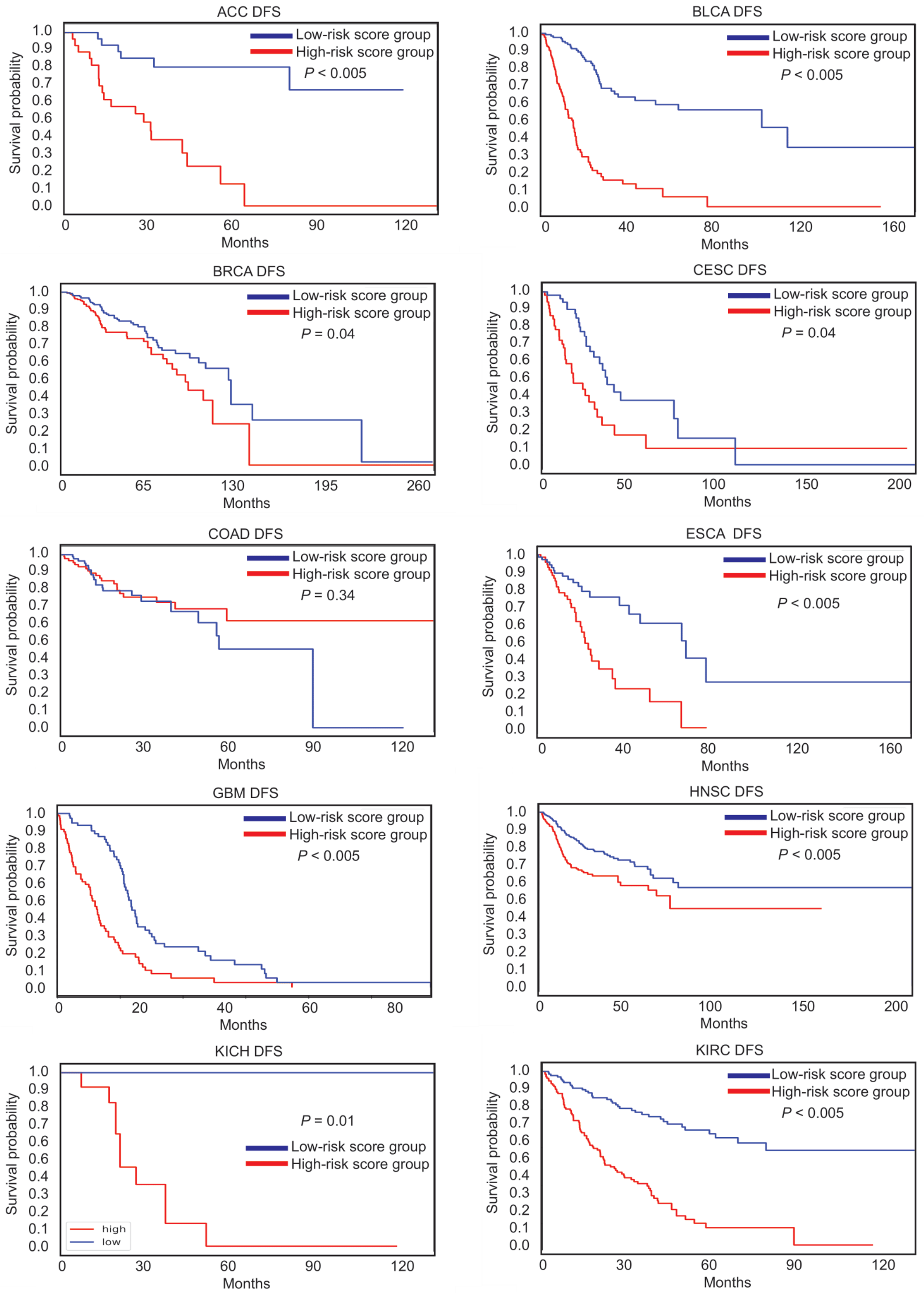

Figure S7 Kaplan-Meier curves for the DFS of patients in the high-risk and low-risk groups for each cancer type (ACC, BLCA, BRCA, CESC, COAD, ESCA, GBM, HNSC, KICH, and KIRC).

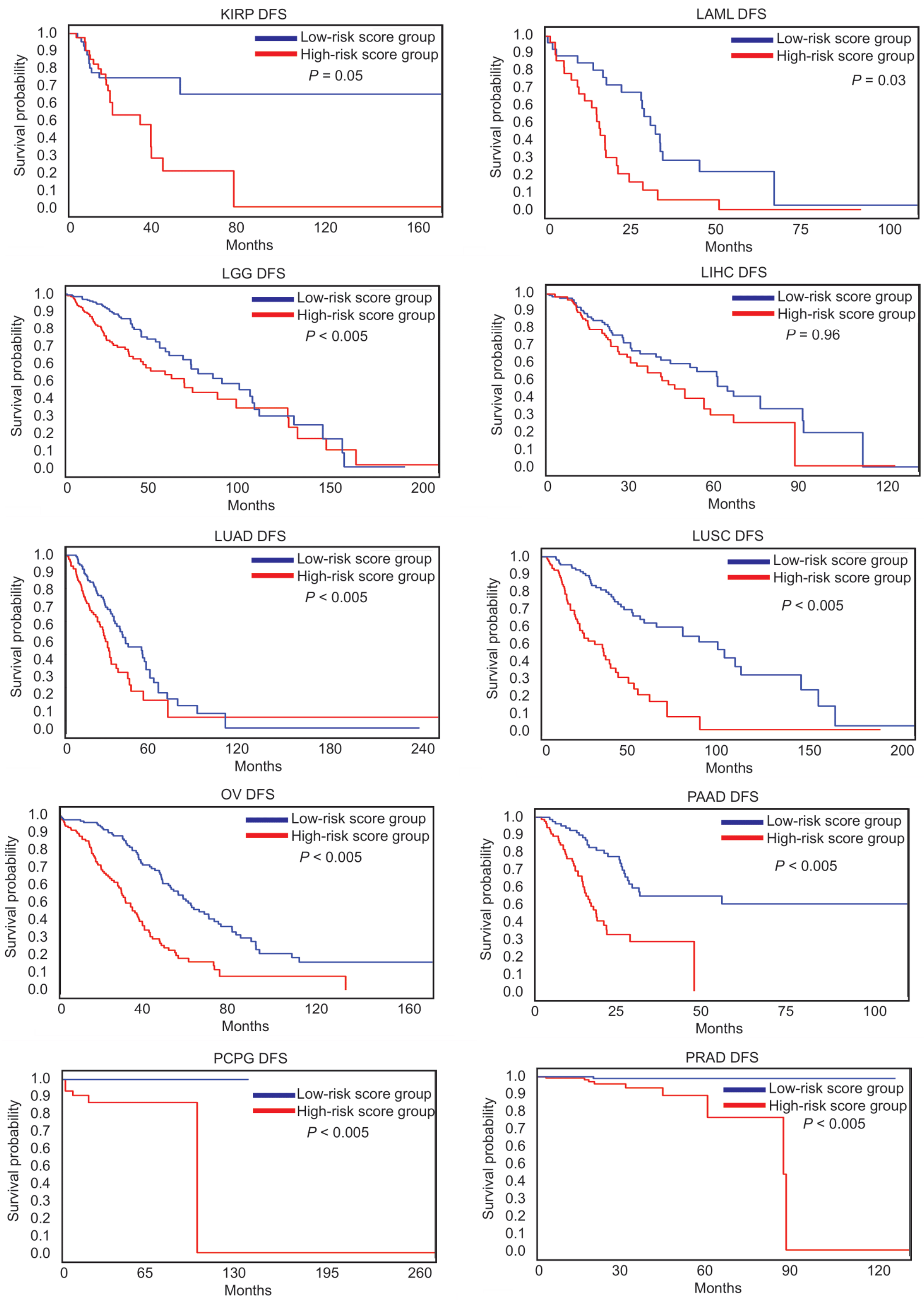

Figure S8 Kaplan-Meier curves for the DFS of patients in the high-risk and low-risk groups for each cancer type (LGG, LIHC, LUAD, LUSC, OV, PAAD, PCPG, and PRAD).

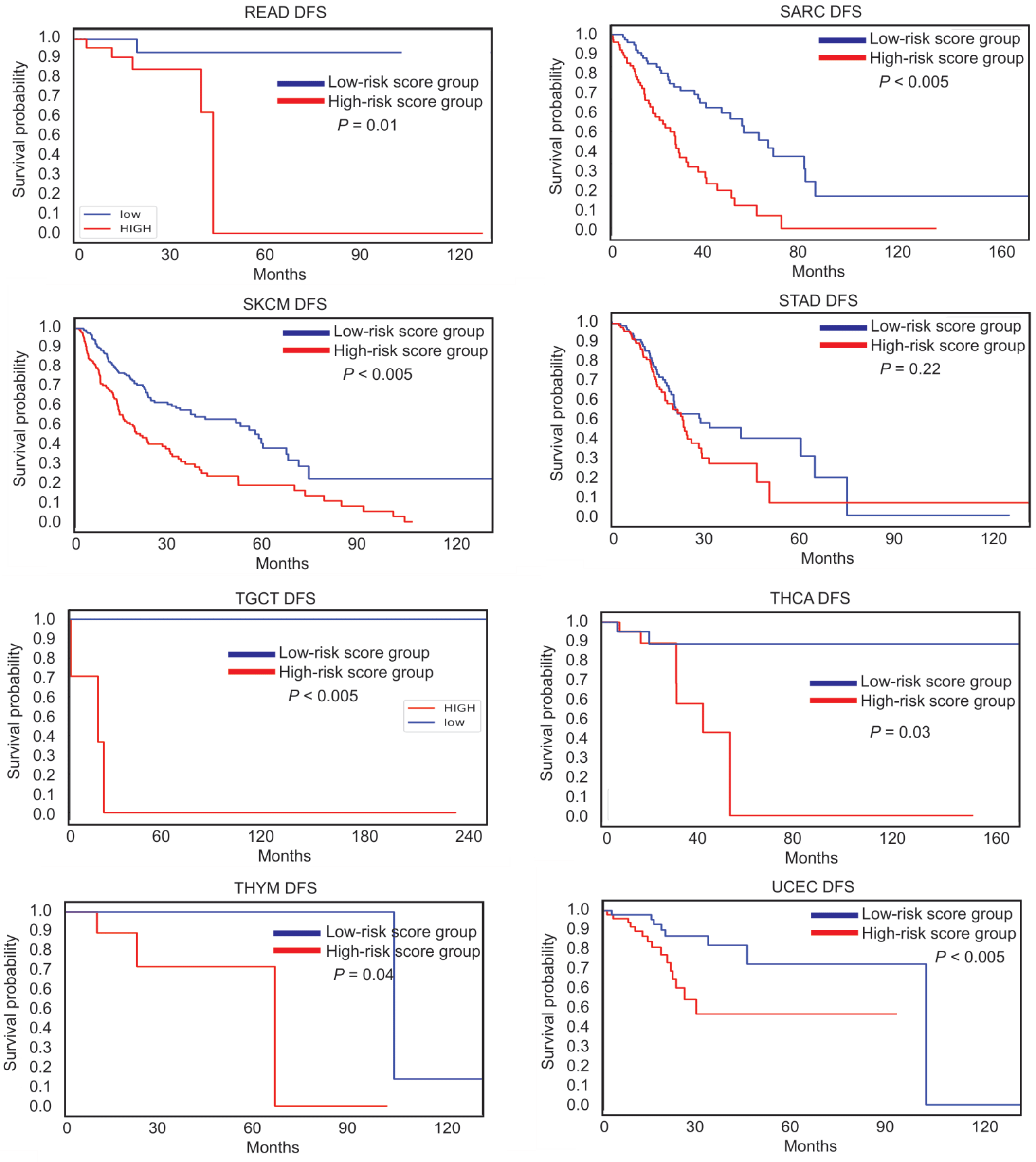

Figure S9 Kaplan-Meier curves for the DFS of patients in the high-risk and low-risk groups for each cancer type (READ, SARC, SKCM, STAD, TGCT, THCA, THYM, and UCEC).

Table S1. Functional classification of genes involved in pyroptosis.

Gene Function	Gene Name
Inflammasome sensor	*AIM2, NLRP1, NLRP2, NLRP3, NLRP6, NLRP7, NLRP9, NLRP12, NLRX1, NLRC4, NOD2, TLR4, MEFV (Pyrin)* (*1, 2*)
Inflammasome adaptor ASC	*PYDC1, PYCARD, CARD8, CARD16, CARD18* (*3*).
Cleavage protease	*caspase-1/3/4/5/8, GZMA, GZMB* (*4*) (*5, 6*).
Pyroptosis executor	*GSDMB, GSDMC, GSDMD, GSDME (DFNA5)* (*7*) (*8-10*) (*11*).
Inflammatory factor	*IL-1β, IL18, HMGB1* (*12*)
Activation of inflammasome	*GBP2*, GBP5, DDX3X, NAIP* (*13, 14*)
The regulators of caspases and GSDMs activation	*SERPINB1, C-FLIP, TNFRSF21, GBP1, GBP2*, GBP3, GBP4, IRF2* (*13, 14*)

* Dual function of *GBP2* in pyroptosis: promoting AIM2 activation during infection with Francisella novicida; and controlling the recruitment of caspase-4 in IFN-γ stimulated cells.

Table S2. The abbreviations, classification and numbers of samples for the 31 cancer types investigated in this study.

Abbreviation	Cancer Type	Number of Cancer Samples	Number of Normal Samples
ACC	Adrenocortical carcinoma	79	17
BLCA	Bladder Urothelial Carcinoma	407	19
BRCA	Breast invasive carcinoma	1097	114
CESC	Cervical squamous cell carcinoma and endocervical adenocarcinoma	303	3
CHOL	Cholangiocarcinoma	36	9
COAD	Colon adenocarcinoma	286	41
DLBC	Lymphoid Neoplasm Diffuse Large B-cell Lymphoma	48	5
ESCA	Esophageal carcinoma	184	11
GBM	Glioblastoma multiforme	154	5
HNSC	Head and Neck squamous cell carcinoma	520	44
KICH	Kidney Chromophobe	66	25
KIRC	Kidney renal clear cell carcinoma	533	72
KIRP	Kidney renal papillary cell carcinoma	290	32
LAML	Acute Myeloid Leukemia	516	110
LGG	Brain Lower Grade Glioma	371	23
LIHC	Liver hepatocellular carcinoma	515	50
LUAD	Lung adenocarcinoma	1017	59

LUSC	Lung squamous cell carcinoma	502	51
OV	Ovarian serous cystadenocarcinoma	304	21
PAAD	Pancreatic adenocarcinoma	178	4
PCPG	Pheochromocytoma and Paraganglioma	179	3
PRAD	Prostate adenocarcinoma	497	52
READ	Rectum adenocarcinoma	94	10
SARC	Sarcoma	259	2
SKCM	Skin Cutaneous Melanoma	104	1
STAD	Stomach adenocarcinoma	415	35
TGCT	Testicular Germ Cell Tumors	150	19
THCA	Thyroid carcinoma	505	59
THYM	Thymoma	120	2
UCEC	Uterine Corpus Endometrial Carcinoma	176	24
UCS	Uterine Carcinosarcoma	57	5

Table S3. The function description of KEGG pathway IDs.

KEGG pathway ID	Function
hsa04664	Fc epsilon RI signaling pathway
hsa04662	B cell receptor signaling pathway
hsa05202	Transcriptional misregulation in cancer
hsa04630	Jak-STAT signaling pathway
hsa04064	NF-kappa B signaling pathway
hsa04015	Rap1 signaling pathway
hsa05340	Primary immunodeficiency
hsa04672	Intestinal immune network for IgA production
hsa04620	Toll-like receptor signaling pathway
hsa05321	Inflammatory bowel disease (IBD)
hsa04612	Antigen processing and presentation
hsa05323	Rheumatoid arthritis
hsa05140	Leishmaniasis
hsa04660	T cell receptor signaling pathway
hsa05322	Systemic lupus erythematosus
hsa04650	Natural killer cell mediated cytotoxicity
hsa04062	Chemokine signaling pathway
hsa05150	Staphylococcus aureus infection
hsa04060	Cytokine-cytokine receptor interaction
hsa04514	Cell adhesion molecules (CAMs)
